# A disconcerting false gastric diverticulum mimicking malignancy

**DOI:** 10.11604/pamj.2019.32.80.18075

**Published:** 2019-02-14

**Authors:** Dhouha Bacha, Asma Sassi, Seifeddine Baccouche, Ghofrane Talbi, Sana Ben Slama, Lassad Gharbi, Saadia Bouraoui, Ahlem Lahmar

**Affiliations:** 1 Department of Pathology, Mongi Slim Teaching Hospital La Marsa, University of Tunis El Manar, Faculty of Medicine, Tunis, Tunisia; 2 Surgery Department, Mongi Slim Teaching Hospital La Marsa, University of Tunis El Manar, Faculty of Medicine, Tunis, Tunisia

**Keywords:** Diverticulum, stomach, surgery, malignancy

## Abstract

Gastric diverticula are the most uncommon form of gastrointestinal diverticula. They can either be of true or false type with different pathogenesis. They may be very challenging to diagnose as symptoms are nonspecific and imaging can simulate a malignant lesion. We report an unusual case of pre-pyloric diverticulum in a 69-year-old man, leading to severe gastric obstruction with a poor general condition. As subsequent endoscopy and imaging were alarming and couldn't exclude malignancy, the patient underwent an antrectomy. The final diagnosis was made on pathological examination. We discuss, through this case, the clinical and pathological features of gastric diverticula with an emphasis on the pathogenesis of this rare entity and the risk of a malignant transformation.

## Introduction

Gastric diverticulum is defined as an outpouching of the gastric wall. It is a rare condition with a reported prevalence of 0.03-0.1% in upper gastrointestinal barium X-Rays, 0.01- 0.11% in endoscopies and 0.03-0.3% in autopsies [1]. They are categorized as congenital (true) and acquired (false) types, of which congenital type is the most common. Gastric diverticula may be a challenging diagnosis as clinical symptoms are nonspecific and radiological findings can simulate a malignant lesion. We report a disconcerting case of a false diverticulum of the pre-pyloric region whose diagnosis was made postoperatively on pathological evaluation.

## Patient and observation

**Figure 1:**
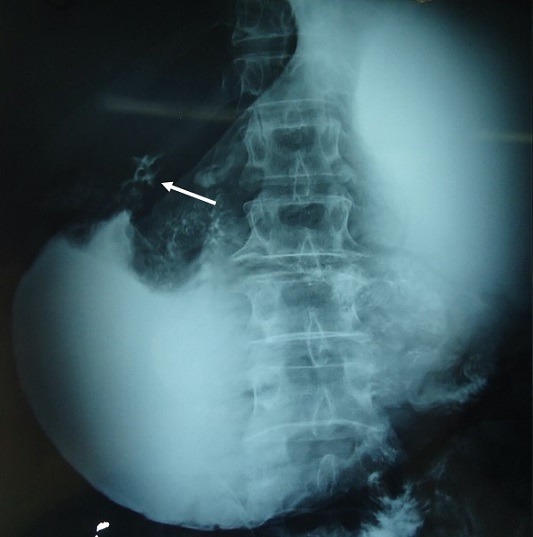
Barium enema: an apple core appearance in the pre-pyloric region

**Figure 2:**
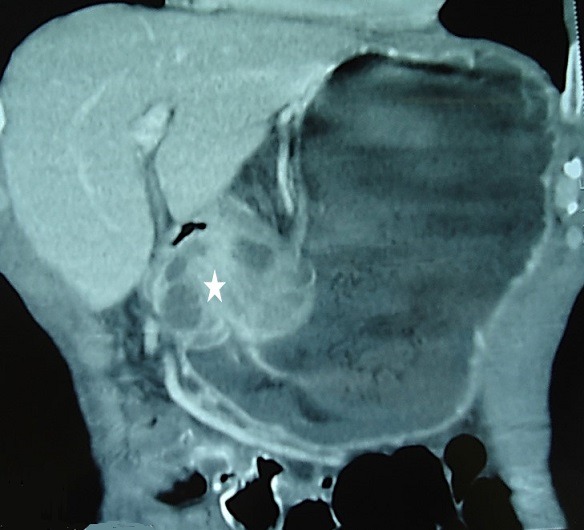
Abdominal CT (coronal view): asymmetric and enhanced wall thickening of the gastric antrum (asterisk)

A 69-year-old man, with no past medical history, presented with a 2-month-history of diffuse abdominal pain and postprandial vomiting. Physical examination revealed a poor general condition with a marked weight loss. The remainder of the examination was normal. Blood investigations were performed and his urea was raised. He maintained a satisfactory haemoglobin level. The upper gastrointestinal endoscopy revealed a distended stomach filled with food residues. The prepyloric region couldn't be crossed. Histological examination of blind biopsy specimens revealed a chronic gastritis with no evidence of dysplasia or malignancy. It was decided to perform an upper gastro-intestinal barium X-Rays, which showed a distended stomach and a very weak passage of the contrast through a stenotic zone in the pre-pyloric region (Figure 1). A subsequent abdominal computed tomography (CT) showed an asymmetric and enhanced 71 x 35mm wall thickening of the pre-pyloric region (Figure 2). This obstruction was resulting in considerable stomach distension. Numerous calcifications were detected throughout the pancreas. Neither focal hepatic lesions nor adenopathy were identified. As it was difficult to make an accurate diagnosis and disprove the likelihood of a cancerous lesion, the patient underwent an exploratory laparotomy. There was no evidence of malignancy on frozen section evaluation. An antrectomy was therefore performed. The patient had an uneventful postoperative course and was discharged from the hospital 7 days later.

**Figure 3:**
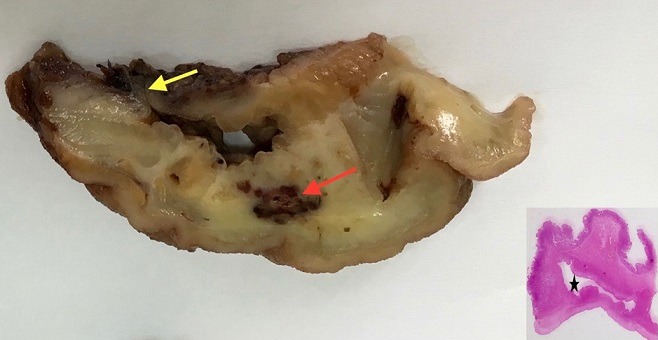
Cut surface of the pre-pyloric region: a diverticulum with a narrow neck (yellow arrow): a brownish lesion adjacent to the diverticulum (red arrow); insert: the diverticulum (asterisk) reaches the muscularis layer on histology (haematoxylin and eosin)

On gross examination, the resected section displayed a wall thickening measuring 70 x 50mm and distant 1.5cm from the pyloric margin. The cut surface of this area was greyish and revealed an underlying 1cm diverticulum with a narrow neck, in diameter and reaching the muscularis layer. A nodular brownish lesion measuring 15 x 10mm and containing distended blood vessels was noticed close by the diverticulum (Figure 3). Tissues were fixed in 10% formalin and embedded in paraffin. Sections were prepared and stained with haematoxylin and eosin (HE). Microscopically, the diverticulum consisted of flask-shaped mucosal outpouching with extension deep into the muscularis layer. The mucosa within the diverticulum mostly showed an ulcerated epithelium that was replaced by a fibrin network. An intense and polymorphic inflammatory infiltrate was present in the diverticular wall with a marked peridiverticular fibrosis. The nodular lesion adjacent to the diverticulum demonstrated abnormally intermixed large, irregular vessels with heterogenic thickened wall. There was no evidence of malignancy. These findings were consistent with the diagnosis of a false pre-pyloric diverticulum with a vascular hamartoma. Six-month follow-up had shown improvement of his general condition with a weight gain of 5 kilos.

## Discussion

Gastric diverticula are the most uncommon form of gastrointestinal diverticula, with a reported prevalence ranging from 0.01 to 0.11% [2]. It occurs equally in men and women, most commonly in the fifth and sixth decades of life [2]. The best diagnostic tools for detecting a gastric diverticulum are upper gastrointestinal barium X-Rays with lateral views and upper endoscopy. Our case was litigious as imaging and endoscopic results have shown a suspicious malignant lesion. We believe that the important fibrosis surrounding the diverticulum and the vascular hamartoma explains well these findings. Gastric diverticula have similar characteristics to their intestinal counterparts. They can be either congenital or acquired with a size ranging from 1 to 11cm [3]. Congenital or true diverticula, comprise 70-75% and are composed of all the layers of the stomach [4]. They are classically located on the posterior part of the cardia and are due to a weakness in the gastric wall. On the opposite, acquired or false diverticula, are much less common and consist in herniation of mucosal and submucosal layers into the muscularis. They are entirely contained within the gastric wall and usually located in the greater curvature of the antrum. In our case, the diverticulum was of acquired type and measured 1cm in diameter.

False diverticula are subdivided into pulsation and traction types. The pulsation type results from conditions associated with high intraluminal pressure or impaired gastric wall as in gastric ulcer, gastric malignancy or prior surgery. The traction type is secondary to perigastric adhesions caused by inflammatory lesions of adjacent organs as the spleen, the pancreas, the liver or the gallbladder [5]. In our case, pancreatic calcifications shown by abdominal CT may suggest an undiagnosed chronic pancreatitis resulting in a traction type of the diverticulum. Gastric diverticula are usually asymptomatic. On occasion, they may become symptomatic resulting in abdominal pain, dyspepsia, vague sensation of fullness, nausea, emesis, and syncope or bowel obstruction as in our case report [6]. Patients may also have a dramatic clinical course and present with gastric perforation, bleeding or malignant transformation [7, 8]. In terms of a possible association of gastric diverticulum with malignancy, only 4 cases in English literature were reported hitherto [9-12]. Such a coexistence is yet to be defined. In most cases, it is more likely to be an inverted type of gastric cancer mimicking a false gastric diverticulum. However, malignant transformation of a gastric diverticulum remains unclear. Although the tumorigenesis of diverticula is controversial, a case report strongly suggests that neoplastic changes in gastric diverticula results from a sequence of progressive metaplasia/dysplasia [11]. Limited histologic data of cancers was provided concerning these case reports (Table 1). Two cases of gastric diverticulum were associated with tubular adenocarcinoma and both of them were pT1a and pT1b according to the WHO classification of tumors of the stomach [11-13]. One case of a gastric adenocarcinoma was arising outside but invading a false diverticulum with a subserosal invasion (pT3) [9]. One early-stage adenocarcinoma was arising in a fundic diverticulum [10]. On the basis of these case reports, we emphasize the importance of performing many samples on gross examination of a gastric diverticulum. That would enable not to overlook carcinoma in or around a diverticulum in microscopic examination.

**Table 1:** Summary of pathological features of malignancy reported in gastric diverticula in the English literature

Authors	Years	Gastric location	Histologic type*	Stage*
Adashi *et al.* [9]	1987	Unspecified	Adenocarcinoma	pT3
Fork *el al.*[10]	1998	Fundus	Adenocarcinoma	Early (unspecified)
Oya *et al.*[11]	2012	Fundus	Tubular adenocarcinoma	pT1a
Lee *et al.*[12]	2016	Antrum	Tubular adenocarcinoma	pT1b

In our case, the main histologic differential diagnosis is a gastric hamartomatous inverted polyp (GHIP), especially that our diverticulum was associated with a vascular hamartoma. This entity has an endophytic growth and is reported on endoscopy as a solitary submucosal mass [14, 15]. Extrusion of milky mucinous material and calcifications from the biopsy site seems to suggest the diagnosis [15]. GHIP is defined as a submucosal proliferation of hypertrophic gastric glands with cystic dilatation [16, 17]. Other pathological characteristics are possible and include smooth muscle proliferation, fibroblastic cells, nerve components and vascular tissue [14]. They are different to authentic hamartomas, which are non-neoplastic formations of intermixed components of the organ originating from [18]. Proper management of a gastric diverticulum depends on its location, its size and symptoms resulting from [3, 19]. There is currently no consensus treatment for asymptomatic diverticula incidentally discovered, which usually don't require any treatment. Symptomatic patients may be treated with H2-receptor blockers or proton-pump inhibitors [20]. Surgical management is considered for large-sized, complicated or distal gastric diverticula as it may be associated with malignancy [12, 19]. Several surgical approaches have been described including invagination of the diverticulum as well as partial gastrectomy [21]. Our patient underwent a partial gastrectomy as a likelihood malignant lesion could not be excluded. The surgical approach was through a median laparotomy or subcostal incision. However, since the first successful laparoscopic resection of a gastric diverticulum in the late 1990s, this approach is now considered as safe and feasible [22].

## Conclusion

We have reported a unique case of a gastric false diverticulum presenting as a suspicious lesion on imaging and endoscopy. Gastric diverticula are usually congenital and asymptomatic. However, false diverticula, especially those located in the prepyloric region, can be associated with malignancy. Pathologists should consider this rare but possible coexistence and do many samples of a gastric diverticulum as well as the surrounding tissue. Appropriate management of diverticula of the stomach depends on size, location, symptoms and complications resulting from. Large-sized, distal and complicated diverticula should be surgically removed either by laparotomy or laparoscopic approach.

## Competing interests

The authors declare no competing interests.
